# A Review of Prolonged Post-COVID-19 Symptoms and Their Implications on Dental Management

**DOI:** 10.3390/ijerph18105131

**Published:** 2021-05-12

**Authors:** Trishnika Chakraborty, Rizwana Fathima Jamal, Gopi Battineni, Kavalipurapu Venkata Teja, Carlos Miguel Marto, Gianrico Spagnuolo

**Affiliations:** 1 Department of Conservative Dentistry and Endodontics, Chaudhary Charan Singh University, Meerut, Uttar Pradesh 250001, India; trishnik@post.bgu.ac.il; 2 Department of Health System Management, Ben-Gurion University of Negev, Beer-Sheva 8410501, Israel; 3 Department of Oral and Maxillofacial Surgery, Chettinad Dental College and Research Institute, Kancheepuram, Tamil Nadu 603103, India; jamriz99@gmail.com; 4 Telemedicine and Tele Pharmacy Center, School Medicinal and Health Products Sciences, University of Camerino, 62032 Camerino, Italy; gopi.battineni@unicam.it; 5 Department of Conservative Dentistry & Endodontics, Saveetha Dental College & Hospitals, Saveetha Institute of Medical & Technical Sciences, Saveetha University, Chennai, Tamil Nadu 600077, India; venkatatejak.sdc@saveetha.com; 6 Faculty of Medicine, Institute of Experimental Pathology, University of Coimbra, 3004-531 Coimbra, Portugal; cmiguel.marto@uc.pt; 7 Faculty of Medicine, Coimbra Institute for Clinical and Biomedical Research (iCBR), University of Coimbra, Area of Environment Genetics and Oncobiology (CIMAGO), 3000-548 Coimbra, Portugal; 8 Centre for Innovative Biomedicine and Biotechnology (CIBB), University of Coimbra, 3004-504 Coimbra, Portugal; 9 Clinical Academic Center of Coimbra (CACC), 3004-531 Coimbra, Portugal; 10 Department of Neurosciences, Reproductive and Odontostomatological Sciences, University of Naples “Federico II”, 80131 Napoli, Italy; 11 Institute of Dentistry, I. M. Sechenov First Moscow State Medical University, 119435 Moscow, Russia

**Keywords:** long-COVID, COVID-19 recovered, prolonged COVID symptoms, COVID-19 dental, post-COVID-19 syndrome

## Abstract

The available data regarding the short and long-term consequences of COVID-19 is still insufficient. This narrative review aims to provide information on the prolonged COVID-19 symptoms in recovered patients and their implications during dental management. Additionally, this manuscript highlights the oral manifestations of COVID-19 and its management. A systematic search was conducted in PubMed, Embase, Cochrane Library and Web of Science databases, WHO and CDC websites, and grey literature was searched through Google Scholar. Clinical articles (clinical trials, case-reports, cohort, and cross-sectional studies) were included, reporting prolonged post-COVID-19 symptoms. Although COVID-19 is an infectious disease primarily affecting the lungs, its multi-organ involvement is responsible for several prolonged symptoms, including oral implications. In recovered patients with prolonged COVID-19 symptoms, considerations for providing dental treatment has to be made as they can present with assortment of symptoms. These prolonged post-COVID-19 symptoms can affect the delivery of the required dental treatment. Hence, the recommendations proposed in this narrative review can be a useful starting point to aid dental teams providing adequate care for such recovered patients.

## 1. Introduction

The novel human coronavirus COVID-19, responsible for the recently named severe acute respiratory syndrome coronavirus 2 (SARS-CoV-2), was first identified in China in December 2019 and turned into a pandemic within a short period [1]. The most common clinical features of COVID-19 include dry cough, fever, dyspnea, myalgia, joint pain, fatigue, gastrointestinal symptoms, and anosmia/dysgeusia [2]. Although the lungs are the first target organ of COVID-19 infection, accumulating evidence indicates that the virus may exhibit infections in different organs, including the heart, blood vessels, kidneys, gut, oral cavity, eyes, and brain [3]. 

According to the World Health Organization (WHO), the mortality rate of COVID-19 patients is 3% to 5%. Reports have suggested that patients who survive COVID-19 may experience impairment or prolonged symptoms in their overall health status after their acute phase recovery [4]. According to the WHO, patients who recover from COVID-19 can have persistent symptoms such as fatigue, dyspnea, dry coughing, congestion or shortness of breath, loss of taste or smell, loss of hearing, body aches, diarrhea, nausea, chest or abdominal pain [5]. Other complications include acute kidney injury with little evidence of renal failure and hepatic impairment in severely ill patients [6]. Moreover, there have been records of changes in the clotting system, such as disseminated endovascular coagulopathy (DIC), decreased platelet count, and prolonged prothrombin time (PT). Additionally, hypercoagulability and potential thromboembolic disorders are a few of these patients’ common manifestations [7,8]. 

Nutritional status is a major health determisant for the recovery of COVID-19 patients, especially in people at risk for adverse outcomes, such as the elderly and those with underlying medical conditions. Previous literature has shown that prolonged intensive care unit stays lead to a decline in muscle mass and strength and anorexia. Moreover, following the COVID-19 infection, malnutrition was aggravated, which was responsible for poor recovery and poor quality of life of discharged ICU patients [9]. Previous reports have associated worse outcomes of COVID-19 patients with low levels of circulating markers of nutritional status [5,9]. Nustrition deficiencies have been acknowledged across all the stages of COVID-19, especially in populations who are at higher risk of negative outcomes [10]. Although this “post-COVID syndrome” may linger or recur for weeks or months following the initial recovery, these recovered patients are not contagious to others during this time. 

Additionally, COVID-19 acute infection, with associated therapeutic measures, could contribute to adverse oral health outcomes. The signs and symptoms in the oral cavity due to COVID-19 are taste disorders, unspecific oral ulcerations, desquamative gingivitis, petechial, and co-infections such as candidiasis [11]. Despite the many studies based on expert opinions on COVID-19 recovery, the clinical picture of the COVID-19 aftermath is still unclear. Dentists, being in close contact with the patient’s droplets and aerosols generated, must revise the operating protocols to protect the team and the patients from the risk of infectious diseases [12]. Since the long-term consequences of the current pandemic are unknown, these circumstances resulted in a “new normal” dentistry provision. 

Several protocols and guidelines for reopening dental clinics and dental treatment have been issued by governments and regional medical and dental authorities, focusing on aerosol reduction and other preventive measures [13]. Studies have concluded that the usage of high-volume evacuators, fumigation using chlorine dioxide and hydrogen peroxide vapor (HPV) are effective for aerosol reduction in dental operating sites [14,15]. Additionally, implementing primary filters, UV light, plasma disinfection, HVAC systems and High Efficiency Particulate Air (HEPA), have resulted in 99.7% air filtration. Currently, researchers are developing protective devices to reduce aerosol dispersion in dental clinics, to prevent COVID-19 transmission [16]. 

However, with the increasing number of recovered COVID-19 patients, needful, and emergency dental care for these survivors would require guidelines that should include an understanding of the persistent symptoms of COVID-19. A detailed analysis of health consequences with longer follow up on COVID-19 survivors, and the duration of its impact is required to provide them with urgent dental care with a multidisciplinary approach. Thereby, this review aims to describe the prolonged symptoms of COVID-19 post-recovery. Additionally, we attempted to summarize the impacts of COVID-19 in dental practice and the possible challenges that dentists might face while treating patients with prolonged symptoms of COVID-19. 

## 2. Materials and Methods

A narrative review was performed focusing on prolonged post-COVID-19 symptoms and implications for dental care. A systematic approach was used to perform the studies identification and selection to increase the review quality and evidence. 

*Aim and Review Questions*

This narrative review aims to summarize evidence on the prevalence of prolonged post-COVID-19 symptoms in recovered patients and the dental management of recovered patients with prolonged symptoms of COVID-19.

This review aims to answer the following specific questions:

1. What are the prolonged post-COVID-19 symptoms in recovered patients?
2. What is the impact of COVID-19 on dental management?
3. What are the clinical implications of prolonged post-COVID-19 symptoms during dental management?
4. What could be the possible challenges for the dental team while treating patients who have prolonged symptoms?

*Inclusion and Exclusion Criteria*

The inclusion criteria consisted of clinical studies on post-COVID-19 prolonged symptoms. Clinical trials, observational studies (cohorts, cross-sectional, case-control) and case reports were considered. 

Regarding the impact of COVID-19 in dental practice and the management of patients with prolonged symptoms of COVID-19, dental guidelines were also screened. 

Additionally, peer-reviewed studies published from January 2020 to 2021 in English were considered.

*Search Strategy*

The literature search was conducted in PubMed (Medline), Cochrane Library, (WoS) databases. An additional search was performed in the WHO and Centers for Disease Control and Prevention (CDC) websites, and grey literature was searched through Google Scholar. A manual search was conducted across the reference lists of the included studies.

The last search was performed on 21 January 2021. 

The search strategy used a combination of the following terms: “COVID-19 recovered persistent symptoms”, “prolonged COVID-19”,” persistent COVID-19 symptoms”, “long term effects of COVID-19”, and “long term symptoms of COVID-19”. 

Regarding the dental management of patients with prolonged symptoms of COVID-19, “oral health”, “dental management”, and “dental procedures and training” were added to the search terms.

*Study Selection*

The studies selection was performed in two phases. In phase 1, two authors independently reviewed the titles and abstracts of all the references through COVIDence software and selected those appearing to meet the inclusion criteria, and the remaining were excluded. In phase 2, the same selection criteria were applied to the full-text articles to confirm those studies, disagreements were resolved through consensus by discussion, and/or arbitration by a third reviewer where required.

## 3. Results

**Figure 1:**
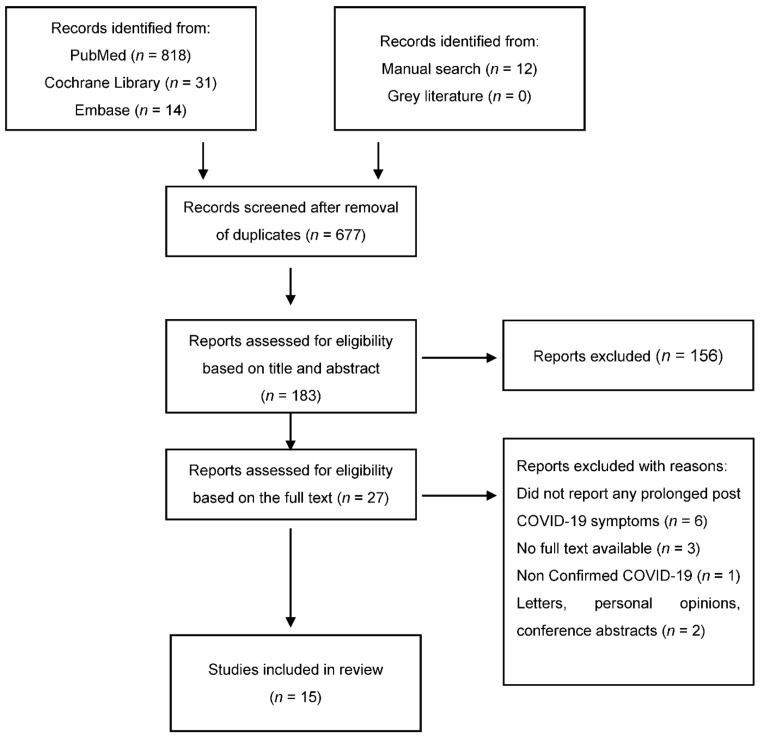
Flow chart of the study selection.

As illustrated in Figure 1, 818 studies were retrieved from PubMed and 54 from the other databases and manual search. COVIDence was used to remove duplicates from a total of 875 articles. Following two stages of screening, a final number of 15 articles were included in the review. Descriptive characteristics of prolonged symptoms in post-COVID patients and associated details are outlined in Table 1. In addition, the impact of COVID-19 in dental management is summarized in Table 2.

### 3.1. Prolonged COVID-19 Symptoms in Post-COVID Patients 

A significant number of post-COVID-19 patients have shown moderate to severe long-lasting impact on their overall health. Although COVID-19 is an infectious disease primarily affecting the lungs, its multi-organ involvement is responsible for several systemic symptoms. One of the most common complications in recovered patients is post-COVID fibrosis or post-ARDS (acute respiratory distress syndrome) fibrosis [17]. Cardiac magnetic resonance imaging of the recovered patients, aged between 40–50, reported heart abnormalities with inflammation signs in the heart muscle. The other findings included stress cardiomyopathy, myocardial injury and acute myocardial infarction (AMI), heart failure, dysrhythmias, and venous/pulmonary thromboembolic events [18]. 

While estimates vary, approximately half of all COVID-19 patients experience neurological symptoms during the acute phase of their illness. Studies have reported that recovered patients manifested altered mental status, headache, seizures, movement disorders, and tremors for months after their recovery. Many post-infectious COVID-19 patients classically manifest with Guillain-Barré syndrome [6]. On the other hand, psychological effects, mostly depression, fear, and anxiety, during the quarantine period and after recovery have been documented among the general population [19]. This can be associated with many factors, such as delayed recovery rate, the stigma of discrimination, financial crisis, prolonged isolation, and limited family engagement [32]. 

Regarding the gastrointestinal system, the most prevalent symptoms are diarrhea, vomiting, nausea, abdominal pain, and/or gastrointestinal bleeding [26]. This is associated with the side effects of drugs, such as antibiotics, antivirals, hydroxychloroquine and biologics, used for treating COVID-19. Notably, many recovered patients reported chronic fatigue syndrome/myalgic encephalomyelitis (CFS/ME), which included symptoms such as persistent fatigue, diffuse myalgia, and psychological issues [20,22,24,27]. This occurs since the virus crosses the blood-brain barrier into the hypothalamus via the olfactory pathway and causes disturbance of lymphatic drainage from the microglia in the brain and accumulation of inflammatory agents [33].

**Figure 2:**
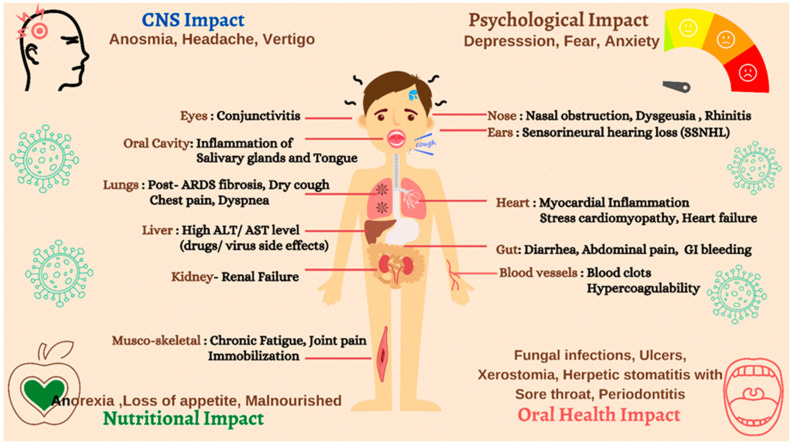
Summary of prolonged post-COVID-19 symptoms in recovered patients.

The case study on otolaryngologic assessment also evidenced nasal obstruction, rhinitis, anosmia, dysgeusia, vertigo, and sudden-onset sensorineural hearing loss (SSNHL) among middle-aged and elderly patients [21]. The COVID-19 infection manifested ocular symptoms such as conjunctivitis, red eyes, swelling of the conjunctiva, overflow of tears onto the face, inflammations and impairment of retinal vascularization [25]. Further, the authors associated the hematological abnormalities, such as increased levels of white blood cells (WBCs) and neutrophils, with COVID-19 patients having ocular symptoms. Additionally, there have been several reports on adverse oral health outcomes, likely leading to various opportunistic fungal infections, such as COVID-19-associated mucor mycosis (CAMCR), hyposalivation causing xerostomia, ulcerations, and HSV-1 due to COVID therapeutic interventions. A summary of these prolonged post-COVID-19 symptoms is illustrated in Figure 2. 

### 3.2. Impact of COVID-19 on Dental Management

The SARS-CoV-2 virus is largely present in nasopharyngeal and salivary emissions of infected patients. Dental professionals may experience patients with suspected or affirmed SARS-CoV-2 disease and should act persistently not exclusively to give care and yet forestall the nosocomial spread of contamination. As of this, hospital admissions for dental infections are limited because the virus spread through saliva [28]. Most dental specialists are not ready to reopen their practices, especially in countries such as Palestine, 60% of dentists produced no confidence to deal with dental patients due to the COVID-19 [30]. 

Previously, studies have reported a low patient count in dental clinics during this public health emergency. The study conducted in Beijing, China supported this statement, due to difficulties in dental emergency management and public health policies, the dental visits decreased significantly [34]. Another cohort study by Taiwan researchers reported a sharp decline of both dental visits and ambulatory care among all healthcare centers, including dental clinics and local hospitals. A high reduction of dental visits appeared at hospitals and emergency medical visits at clinics. However, in the post-COVID-19 period, there can be a sudden surge of dental due to limited procedures during the pandemic [31,34].

## 4. Discussion

The present article reviews the prolonged symptoms of COVID-19 among recovered patients and their implications for dental management. The emergence of the COVID-19 pandemic led to the development of many dental practice guidelines and the inclusion of respiratory hygiene/cough hygiene as a Standard Precautions component. However, there is a need to adapt the guidelines for patients with prolonged post-COVID-19 symptoms to avoid risks and complications during dental procedures in these patients. Health care professionals, especially dental teams, should be well-prepared to manage these recovered patients pragmatically and symptomatically, emphasizing holistic support.

### 4.1. Implications of COVID-19 on Online Triaging and Patients Management

A vast number of studies have reported tele dentistry as an emerging paradigm during this pandemic [35]. Modified structured telephone and online triaging should be implemented with an artificial intelligence-based dental screening software, such as Dental Monitoring and Smile Mate. Virtual consultations using these screening tools present advantages, such as building rapport with the patients before their first clinical appointment, improving patient care and engagement, and reducing unplanned appointments [36]. Home monitoring and follow-ups of these patients are essential for collecting data.

These COVID-19 recovered patients should be treated with utmost care and empathy. The family members should accompany these patients to monitor the dentists’ instructions at home. Patients should be scheduled according to their risk status. Appointments for high-risk patients should be scheduled at the end of a morning shift or at the end of an evening shift to minimize interaction with other patients in the waiting room. It is mandatory to avoid several patients in the waiting room, maintain social distancing of at least 2 m and face masks. Additionally, it is suggested that patients should not carry their personal belongings in the dental operative room. A summary of the proposed health assessments and dental setting guidelines for the treatment of COVID-19 patients is presented in Table 3.

### 4.2. Dental Treatment Considerations for Post-COVID-19 Patients with Prolonged Symptoms

The prolonged COVID-19 symptoms previously described present a challenge in dental care since these patients present a higher risk for oral diseases and/or higher risk for dental care associated complications, as illustrated in Table 4. 

Several studies have reported cardiovascular and vascular complications in one-fourth of the patients hospitalized with the infection [37]. When prolonged-COVID patients with bleeding disorders develop pain associated with irreversible pulpitis or necrosis, a root canal treatment should be considered over-extraction in terms of the potential risk of bleeding. Additionally, bleeding can be limited by calcium hydroxide intra-canal dressing and irrigation with a sodium hypochlorite solution. Previous evidence has suggested infiltration from the mouth’s vestibule side as a safe anesthetic administration for patients with hemophilia, so it can also be used in COVID-19 patients. Post-COVID-19 patients should be prescribed paracetamol (acetaminophen) at a dose not exceeding 60 mg/kg/day or 3 mg/day over nonsteroidal anti-inflammatory drugs (NSAIDs), as paracetamol does not increase bleeding or influence platelet aggregation. If extraction is the last option, post-extraction instructions should include avoiding NSAIDs and maintaining a pressure tampon for more than 2 h after the extraction. Resorbable sutures should be used, and hemostatic agents can be considered to prevent secondary bleeding [38,39]. Local and systemic measures of bleeding control according to ASH or ISTH-IG guidelines could be followed in cases of intraoperative bleeding in these patients [39]. 

Additionally, thromboprophylaxis should be considered. Hence, consultation with the cardiac physician before planning the dental procedure is vital. In patients with confirmed COVID-19 infection, several changes in the coagulation system, such as a hypercoagulable state, were reported. Using warfarin, heparin-based therapy, and antiplatelet drugs for anticoagulation therapy, prolonged thromboprophylaxis is part of the therapeutic intervention for these patients [40]. Previous reports have suggested that unless there are signs of complications, such as pleural inflammation or superinfections, a cough can be best managed by breathing exercises [41]. The most efficient management of chronic cough in dental care would be directing the patient to sit in a supported position and breathe in through the nose and out through the mouth slowly, while relaxing the chest and shoulders and allowing the tummy to rise. The chair position while performing the treatment should be in the upright or semi-supine position [42]. Antitussives or lozenges can be administered for cough suppression. 

### 4.3. Oral Manifestations of COVID-19 and Its Management

Several studies of COVID-19 reported high prevalence of gustatory dysfunction, xerostomia, sialadenitis, and inflammatory reactions in the salivary glands and tongue. The literature also reported various opportunistic fungal infections, ulcerations, and HSV-1 infection due to COVID-19 therapeutic interventions. In recent literature, fungal infections typically Mucor mycosis has been reported in post-COVID patients [43]. It is attributed to occur as a consequence of steroid therapy and in uncontrolled diabetics. Other reports have shown secondary infections such as gingivitis and periodontitis due to dysregulated inflammatory response and cytokine storm. Furthermore, reports of painful herpetic recurrent stomatitis on the palate accompanied by sore throat, blisters on internal labial mucosa with desquamative gingivitis, necrotic interdental papillae with unprovoked gingival bleeding, ulcers on tongue, erythematous lesions and erosions on lips and buccal mucosa exist [44,45,46,47]. Due to a lack of evidence on pharmacological interventions against COVID-19, oral and systemic multidrug-resistant infections can be a potential challenge to treat. However, recent studies [46,47] attempted to classify the oral manifestations of COVID-19 based on the features of the lesions, the timing of presentations, and the therapies into the following:

- Probably pre-existing conditions: Geographic tongue, fissured tongue.
- Sars-CoV-2-related lesions: Early ulcerative lesions, blisters, early erythema multiforme-like lesions, and petechiae.
- Treatment-related lesions: Late ulcerative lesions, late erythema multiforme-like lesions, Candidiasis, angina bullosa, spontaneous oral hemorrhage, and petechiae.
- Lesions related to poor oral hygiene: Ulcero-necrotic gingivitis.

The following topical management of oral diagnosis can be administered:

1. Hyaluronic acid gel and chlorhexidine 2% mouthwash or gel (twice a day) for 14 days in patients with ulcero-erosive lesions [48,49];
2. Miconazole Nitrate twice a day in patients with cytological diagnosis of candidiasis.
3. Tranexamic acid for local hemorrhages [50].
4. Previous studies have suggested a biopsy in the absence of healing after 14 days among mild COVID-19 patients with prior rinsing with chlorhexidine 2% mouthwash for at least 1 min.

It is recommended to perform an extensive intraoral examination in recovered COVID-19 patients to find any related oral manifestation [51]. Dentists should have a high degree of clinical suspicion and keep COVID-19-associated Mucor mycosis (CAMCR) in the differential of a severely ill patient with COVID-19 and diabetes mellitus, especially if rhino-orbital or rhino-cerebral presentations are noted. Additionally, the dentist should examine the salivary glands and saliva flow to perform early diagnoses related to changes in the glandular parenchyma that might be affected by the virus.

Table 3 summarizes the clinical conditions associated with the prolonged COVID-19 symptoms and the suggested dental recommendations and management. 

### 4.4. Dental Implications of Prolonged COVID-19 Symptoms in Geriatric Dentistry

Elderly patients who have survived are at high risk of sarcopenia, malnutrition, depression, and delirium. This poses a challenge for the dental team to perform relationship-based care for patients with complex needs. Atypical presentation of fatigue and musculoskeletal weaknesses for a prolonged duration in elderly individuals poses a challenge for dentists to complete complex dental procedures in a short period and with fewer appointments. The dental fear and anxiety can be categorized under “The Seattle System”, and as per the guidelines, dental anxiety can be managed [51,52,53]. Psychotherapeutic interventions such as Ost’s applied relaxation technique, Jacobsen’s progressive muscular relaxation, functional relaxation, rapid relaxation technique, and autogenic relaxation can also be used to relieve stress, especially for uncooperative patients [53,54]. Hence, patients’ scheduling and triaging are critical parameters to be managed before re-starting the dental setup. 

It will be challenging for patients who suffer from chronic cough to treat with rubber dams and perform extractions and other complex dental treatments, especially in elderly patients. 

Despite the advantages of tele dentistry, setting up an online follow-up for elderly patients with cognitive impairment or dementia and mental illness (for instance, depression, anxiety) will be challenging. Last, accessibility to elderly patients residing in long-term care institutions is a barrier, as caregivers are generally limited and poorly trained to address oral health care.

While the current guidelines are a helpful starting point to move our practice forward, they should be expanded to include the post-COVID-19 patients, many with prolonged symptoms [55,56]. Future research should be carried on the long-term effects of the COVID-19 virus in the oral cavity and the upper aerodigestive tract, effects of thromboprophylaxis in emergent dental treatment, and safe administration of perioperative antibiotics and analgesics in post-COVID-19 patients. 

This will help dental teams in better preparedness and management of recovered COVID-19 patients with prolonged symptoms. 

## 5. Conclusions

Although COVID-19 is an infectious disease primarily affecting the lungs, its multi-organ involvement requires attention while planning dental procedures for post-COVID patients with prolonged symptoms. 

An appropriate identification and management of prolonged COVID-19 symptoms by the dental team will reduce the risk for dental treatments associated complications and provide better dental care for these patients. 

This review synthesized the available evidence regarding:

- The lesions in oral mucosa in long COVID patients due to various therapeutic approaches of COVID-19.
- The impact of prolonged symptoms in post-COVID-19 patients on urgent dental care.
- Dental considerations and clinical management of COVID-19 recovered patients.
- Implications of online triaging post-COVID-19 patients.
- Classifications of oral manifestations of COVID-19 and its management.
- Implications of AI-based dental screening software in geriatric consultations.

## Figures and Tables

**Table 1:** Summary findings of prolonged post-COVID-19 symptoms.

Author and Year	Sample	Study Type	Prolonged Symptoms of COVID-19
Carfi et al., 2020 [17]	143	Case Series	Most common symptoms including fatigue, dyspnoea, joint pain, chest pain, cough, anosmia. Less common symptoms include Sicca syndrome, rhinitis, dysgeusia, headache, sputum production, vertigo, loss of appetite, sore throat, myalgia, diarrhea.
C D Rio et al., 2020 [18]	292	Cross sectional	Cardiovascular: Myocardial inflammation and myocarditis, and cardiac arrhythmias. Pulmonary: Interstitial thickening and fibrosis, decreased diffusion capacity for carbon monoxide, abnormalities in pulmonary function test, decreased diffusion capacity for carbon monoxide, and diminished respiratory muscle strength. Neurologic: Headache, vertigo, and chemosensory dysfunction, stroke, encephalitis, seizures, mood swings, and brain fog. Emotional health and well-being feelings of isolation and loneliness, COVID-19–related stigma, lingering malaise and exhaustion akin to chronic fatigue syndrome, depression, anxiety, posttraumatic stress disorder, and substance use disorder.
E Garrigues et al., 2020 [19]	120	Cross sectional	Fatigue, dyspnoea, loss of memory, concentration and sleep disorders, ageusia, anosmia, hair loss, memory loss.
Halpin et al., 2020 [20]	100	Cross sectional	Fatigue, breathlessness, persistent cough, concentration problems, post- traumatic stress disorder (PTSD), voice changes, anxiety, depression, continence problems, memory problems, dysphagia.
Koumpa et al., 2020 [21]	45-year-old patient	Case report	Sudden onset sensorineural hearing loss (SSNHL).
Moreno-Perez et al., 2021 [22]	277	Cohort study	Fatigue, dyspnoea, anosmia, amnesic complaints, cough, dysgeusia, headache.
Amorim dos Santos et al., 2020 [23]	77-year-old male patient	Case report	Fungus infection, Herpetic recurrent oral lesion, Fibroma and geographic tongue as a result of COVID-19 specific treatment.
R Perrin et al., 2020 [24]	42-year-old male patient	Case report	CFS/ME symptomatology such as persistent fatigue, diffuse myalgia, depressive symptoms, and non-restorative sleep.
A Sardasi et al., 2020 [25]	31-year-old patient	Case report	Myocarditis due to residual myocardial inflammation.
Wang et al., 2020 [26]	131	Cohort study	Cough, fatigue, expectoration, chest tightness, dyspnoea, chest pain, dizziness, palpitation. Other rare symptoms, including pharyngeal pain, nausea, inappetence and vomiting.
Weerahandi et al., 2021 [27]	161	Cohort Study	Dyspnoea, altered mental status.

**Table 2:** The impact of COVID-19 in dental management.

Author and Year	Sample	Study Type	COVID-19 Impact on Dental Management
Samara et al., 2021 [28]	11	Cross-sectional	The number of hospital admissions for cervicofacial infections decreased by 35% during the COVID-19 pandemic when dental practices were closed. There was an increase in cases treated with intravenous antibiotics and extraction under local anesthetic in 2020. The mean CRP during the period of lockdown was significantly higher compared to the same period 1 year ago.
Petrescu et al., 2020 [29]	884	Cross-sectional	Acute apical periodontitis (42.3%), acute pulpitis (33.3%), and cellulitis/abscess (9.3%) were the most frequent diagnosis. The percentage of patients receiving sedative filling for acute pulpitis treatment in 2020 was significantly higher than in 2019. Dental emergencies were higher for the age group of 20–50 years. The most frequent treatment performed for the abscess was endodontic drainage, both in 2020 and 2019.
Kateeb et al., 2021 [30]	488	Cross-sectional	Almost 13% reported a lack of confidence in dealing with patients with COVID-19, while 64% had “little to moderate” confidence. Most dentists (88%) preferred not to treat patients with COVID-19, while 40% were willing to provide care. 75% reported financial hardships due to the pandemic. 61.2% of the participants felt confused about the protocol’s procedures, while 78% demanded updating of the current protocol to reopen dental clinics to routine care.
Lee et al., 2021 [31]	6681 medical visits	Cohort	The highest reduction in ambulatory medical visits was at clinics, while the most severe dental visits reduction was at hospitals. Due to the postponement of non-emergency or highly infectious dental procedures, the investigators anticipate that more severe dental problems or complications may occur in the post-COVID-19 period.

**Table 3:** Suggested health assessments and dental setting guidelines for the treatment of COVID-19 patients.

Dental Care Phases	Suggested Evaluation and Dental Setting Guidelines
Primary Teledentisty Examination	Ask the patient to upload intra oral pictures in different perspectives using phone camera and tablespoons in the SmileMate
	Ask for the medical and medication history
	Ask the patient about the past and present signs and symptoms of COVID-19
	Ask the patient about the treatment received for COVID-19 (supplemental oxygen, antibiotics, anti-retroviral, HCQ, immunomodulators)
	Check the past diagnostic reports of COVID-19
	Share the comprehensive dental report based on Online dental screening software (SmileMate) with the patient
	Patient counselling and treatment recommendation should be advised
Comprehensive COVID-19 post-acute assessment	Oxygen saturation, Heart rate, Blood pressure assessment
	Lifestyle assessment (physical activity, diet, alcohol consumption)
	Ask for gastrointestinal symptoms
	Physical perfomance test (6 min walking, hand grip and chair side stand) for the elderly patients
	Psychiatric history and quality or life assessment
Dental facility considerations for COVID-19 recovered patient	The appointments for the patients who have persistent symptoms should be preplanned (either first or the last appointment)
	Shorter waiting time
	Mandatory use of facemasks in the waiting room
	Waiting area should allow social distancing (6-feet/2 m) apart
	Provision for tissue paper dispenser and foot operated waster bin
	Use of HEPA filters in dental care facilities with commercial split and centralized/window Acs
	Proper ventilated dental operatory rooms
	Administer frequent disinfection of touched surfaces with NaOCl and ethanol
	Disinfecting the floors or the operatory room with 1000 mg/L chlorine
	’Critical’ heat sensitive instruments should de disinfected with 2 %glutaraldehyde
	Waste disposal in accordance to the CDC guidelines
Dental radiography	Extraoral radiography (panoramic radiography or cone-beam CT)
Succesive follow-ups	Providing the patient with cheek-retractors
	Regular follow-ups by using oral health assessment forms or SmileMate monitoring

**Table 4:** Clinical considerations of post-COVID-19 patients and suggested dental management.

Clinical Consideration	Clinical Condition/Situation	Suggested Dental Recommendations and Management
Respiratory	Breathlessness	Periodic recording of oxygen saturation for a week by the patient prior to treatment
		Continous monitoring of oxygen saturation by "pulse oximeter" during the treatment
		Practice and train breathing techniques (inspiration to expiration ratio of 1:2)
		Bilateral mandibular blocks should not be administered
		Clinics must include medical emergency first aid kits (oxygen cylinders)
	Cough	Practice and train breathing techniques (inspiration to expiration ratio of 1:2)
		Antitussives or lozenges for immediate cough suppression.
		Chair position during the treatment: Upright or semi supine position
Psychosocial	Fear in COVID-19 recovered pts.	Virtual consultations using AI based patient management screening tools
	Fear and Anxiety in COVID-19 recovered pts	Appointments to be scheduled after complete health assessment
		Family members should also accompany during the appointment.
		First or last time slot should be scheduled
		Screen the patients using "The Seattle System for anxiety and fear
		To be treated with utmost care and empathy
	Stress in COVID-19 recovered pts.	Psychotherapeutic interventions can be used
	Fear in Dentists	Learning about the virus and post-COVID symptoms
Oral health	Inflammatory reactions (salivary glands, tongue)	Dental follow-ups of recovered patients
	Pain	Acetaminophen (not exceeding 60 mg/kg/day or 3 mg/day)
	Periodontal	Oral health hygiene training (online, if necessary)
		Regular online follow ups (patient management software can be used)
Musco-skeletal	Associated sleeplessness and anxiety	Non-pharmacological (3 ps Technique by RCOT)
		Pharmacological interventions (tranquilizers, muscle relaxants or anxiolytics)
	Fatigue	Pre-planning the treatment
		Short appointments and relaxing setting
Bleeding disorders	Active Bleeding	ASH guidelines for controlling bleeding (who are not under thromboprophylaxis)
Bleeding disorders	Pain	Acetaminophen (not exceeding 60 mg/kg/day or 3 mg/day)
(Hypercoagulability thromboembolic disorders/Congenital Bleeding Diathesis) cardiac Damages (Stress cardiomyopathy)	Pain due to Irreversible Pulpitis/necrosis	Endodontic treatment should be considered over extraction
		Endodontic consideration: Copious irrigation with sodium hypochlorite sol.
		Endodontic consideration: lntracanal dressing to limit the bleeding from canals
		Surgical consideration: Short appointments
		Safe anaesthsia: Infiltration from the vestibule side of the mouth
		Surgical consideration: Resorbable sutures and haemostatic agent to be used
		Instruction to patient.: Maintain a pressure tampon for 1–2 h after extraction
